# Supplementary material for: Bacterial and fungal communities respond differently to varying tillage depth in agricultural soils
Source: PeerJ. 2017 Oct 17;5:e3930. doi: 10.7717/peerj.3930 (PMC5649590; doi:10.7717/peerj.3930)
Supplement: Figure S1 — The interaction terms for (h) exchangeable acidity and (i) exchangeable aluminium were non-significant. Data are (dark blue) Permanent fallow; (light blue) Permanent pasture; (black) intensive tillage; (red) Moderate tillage; (green) No tillage. [file peerj-05-3930-s004.pdf]

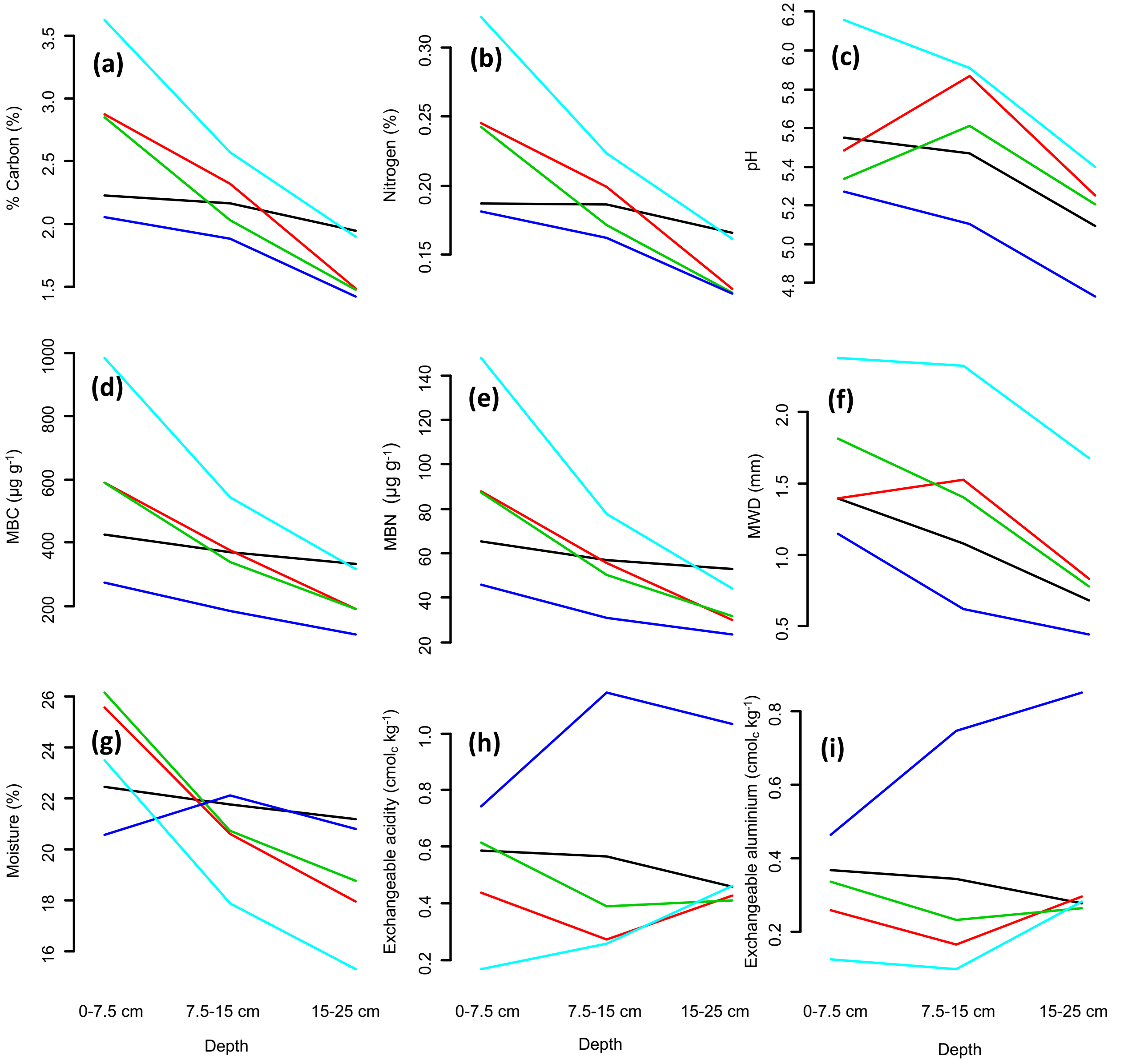

Supplementary Figure 1. Two-way interaction plots for soil chemical variables showing the effects of treatment and depth. The interaction terms for (h) exchangeable acidity and (i) exchangeable aluminium were non-significant. Data are (dark blue) Permanent fallow; (light blue) Permanent pasture; (black) intensive tillage; (red) Moderate tillage; (green) No tillage.
